# Audit of Adherence to Prescribing Guidelines of Psychotropic Drugs for People With Intellectual Disability and Behaviours That Challenge -Specialist Support Team -SST-Merseycare NHS Foundation Trust

**DOI:** 10.1192/bjo.2022.441

**Published:** 2022-06-20

**Authors:** Syeda Hasan, Mark Spurrell

**Affiliations:** 1 Pennine Care NHS Foundation Trust, Manchester, United Kingdom; 2 Mersey Care NHS Foundation Trust, Manchester, United Kingdom

## Abstract

**Aims:**

There are concerns following the Winterbourne view investigation and from the Learning Disabilities Census that psychotropic medications are being inappropriately given to people with learning disability as a means of managing difficult behaviours. Stopping Overuse of Medication in People with Learning Disability (STOMP) is a key area which has been identified as needing improvement for the Transforming Care Programme which is being supported by the Royal College of Psychiatrists. Members are encouraged include STOMP in their local audits. It is for this reason that the topic has been chosen. The overall aim of this project is to capture the snapshot of prescribing of psychotropic medications for people under SST care. This information has been used for establishing baseline of current practice as they are happening and to develop SST base response to support STOMP agenda.

**Methods:**

The population audited was patients open to the SST LANCS/GM. Patients had to be between 18 and 65 years old, have a diagnosis of a learning disability and be known to have challenging behaviour. Patients were excluded from the audit if they had no challenging behaviour, had been discharged from services. The sample size was 20 (10 from GM and 10 from LANCS). Data were collected using the proforma and then entered into Microsoft excel for analysis.

**Results:**

Four overall standards were audited, each with key lines of enquiry within the standard audited to help determine compliance. Overall compliance for standard one, the indication and rationale should be clearly stated, was 50%. For standard 2, consent to treatment procedure, the third standard, regular monitoring of the treatment response and side effects, and the final standard, review and evaluation of the need for continuation or discontinuation of the psychotropic drug, the compliance was less than 10%. It should be noted that the audit erred on the side of counting in any information that suggested that the theme had been thought about at all: an in depth capturing of any of the themes did not feature in the sample.

**Conclusion:**

This audit highlights important issues for improvement within the SST both in relation to better supporting STOMP and good psychotropic medication management practice, and in relation to its documentation. It also serves as a springboard to a number of initiatives that would help to turn that situation around. In the light of concerns, an early re-audit of practice is recommended.

